# MRI resting-state signature of the propensity to experience meaningful coincidences: a functional coupling analysis

**DOI:** 10.1093/cercor/bhae269

**Published:** 2024-07-10

**Authors:** Christian Rominger, Karl Koschutnig, Andreas Fink, Corinna M Perchtold-Stefan

**Affiliations:** Institute of Psychology, University of Graz, Universitätsplatz 2/III, A-8010 Graz, Austria; Institute of Psychology, University of Graz, Universitätsplatz 2/III, A-8010 Graz, Austria; Institute of Psychology, University of Graz, Universitätsplatz 2/III, A-8010 Graz, Austria

**Keywords:** apophenia, synchronicity, resting-state connectivity, seed-to-voxel, fc-MVPA

## Abstract

The propensity to experience meaningful patterns in random arrangements and unrelated events shows considerable interindividual differences. Reduced inhibitory control (over sensory processes) and decreased working memory capacities are associated with this trait, which implies that the activation of frontal as well as posterior brain regions may be altered during rest and working memory tasks. In addition, people experiencing more meaningful coincidences showed reduced gray matter of the left inferior frontal gyrus (IFG), which is linked to the inhibition of irrelevant information in working memory and the control and integration of multisensory information. To study deviations in the functional connectivity of the IFG with posterior associative areas, the present study investigated the fMRI resting state in a large sample of *n* = 101 participants. We applied seed-to-voxel analysis and found that people who perceive more meaningful coincidences showed negative functional connectivity of the left IFG (i.e. pars triangularis) with areas of the left posterior associative cortex (e.g. superior parietal cortex). A data-driven multivoxel pattern analysis further indicated that functional connectivity of a cluster located in the right cerebellum with a cluster including parts of the left middle frontal gyrus, left precentral gyrus, and the left IFG (pars opercularis) was associated with meaningful coincidences. These findings add evidence to the neurocognitive foundations of the propensity to experience meaningful coincidences, which strengthens the idea that deviations of working memory functions and inhibition of sensory and motor information explain why people experience more meaning in meaningless noise.

## Introduction

Most of us might have experienced meaningful coincidences such as receiving a phone call from a friend we have not met for a long time, precisely when we are thinking about this friend. This phenomenon is also termed synchronicity, which is characterized by a sudden perception of a significant connection between objectively unrelated events, which provokes a strong feeling that the thought about the friend (i.e. inner event) somehow causes the phone call of this friend (i.e. the outer event; see, e.g. Diaconis and Mosteller 1989). This example illustrates the fundamental connection between the experience of meaningful coincidences and contingency learning as a relevant mechanism of human information processing (see, e.g. Rominger et al. 2011).

People show considerable individual differences in the frequency with which they experience meaningful coincidences (Bressan 2002; Rominger et al. 2011). In general, the propensity to experience meaningful coincidences appears maladaptive, as it is associated with paranormal beliefs, positive schizotypy, and apophenia as a prodromal syndrome of schizophrenia (Conrad 1958; Brugger et al. 1995; Bressan 2002; van Elk et al. 2016), depressive symptoms (Russo-Netzer and Icekson 2022), and reduced working memory capacities (i.e. higher proactive interference; Rominger et al. 2011, 2019). Neuroscientific research studies mirrored the alterations in cognitive functioning by reporting deviations in brain functioning during (auditory) sensory perceptions (Rominger et al. 2018), working memory tasks (Rominger et al. 2019), and resting-state conditions (Rominger et al. 2023), as well as altered brain structure (Unger et al. 2021). Notably, the reduced skill to manipulate content in working memory (keeping relevant information in and irrelevant information out of memory; i.e. working memory hypothesis) as well as lower control over sensory perceptions (i.e. perceptual alteration hypothesis) are two main neurocognitively driven hypotheses explaining why some people experience more meaningful coincidences than others (Diaconis and Mosteller 1989; Rominger et al. 2011, 2018).

Hintzman et al. (1978) suggested that since people unconsciously and continuously track their (internal and external) environment, they may perceive coincidences when a current event matches retrieval cues of an irrelevant event in memory. Following this working memory hypothesis, Rominger et al. (2011) found that people experiencing more meaningful coincidences showed increased proactive interference, which is the conflict of older information with newer information (e.g. retrieval cues) in working memory. An EEG study replicated this finding (Rominger et al. 2019). The authors concluded that frontal control mechanisms may fail in people who experience more meaningful coincidences, while their working memory is under a high load. Interestingly, fMRI studies indicated that the proactive interference effect is more prominent when activation of the (left) inferior frontal gyrus (IFG), specifically the pars triangularis, is lower (Badre and Wagner 2005; Jonides and Nee 2006; Nelson et al. 2009). Since people experiencing more meaningful coincidences have deficits in keeping relevant information in, and irrelevant information out of working memory, the IFG and its functional coupling might consequently show deviations in people high in coincidence perception (Rominger et al. 2011, 2019).

In line with this argument, Unger et al. (2021) reported less gray matter volume in the left IFG (i.e. pars opercularis), in areas of the left parietal association cortex (i.e. superior/inferior parietal cortex) as well as the left medial prefrontal cortex in women with a higher propensity to experience meaningful coincidences. This finding further strengthens the idea of reduced inhibitory skills and reduced working memory capacities in people perceiving more meaningful coincidences (Rominger et al. 2011).

Further relevant to the present study, Rominger et al. (2023) reported more activation of inhibitory brain mechanisms (i.e. higher alpha power increases; see e.g. Fries 2005; Klimesch et al. 2007; Jensen and Mazaheri 2010 for gating by inhibition) in people high in coincidence perception, which was already present under low working memory load (i.e. during an eyes-closed resting condition vs. eyes open). In line with the perceptual alteration hypothesis, this finding signals the failure of control mechanisms crucial to inhibit irrelevant (sensory-based) information during a resting-state condition (i.e. eyes closed) in people experiencing more meaningful coincidences. Similarly, research indicated heightened neuronal processes as response to auditory stimuli in perceivers (i.e. higher auditory evoked N1 amplitude; Rominger et al. 2018). In conclusion, this pattern of findings argues for deviations in brain functioning important to control and inhibit irrelevant (sensory) information from the environment, which in turn might increase the chance to perceive random events as significantly connected.

From a state-of-the-art neuroscientific perspective, these inhibitory functions relevant for sensory processing and working memory emerge from a complex interplay between brain areas, represented in their functional connectivity. However, to the best of our knowledge, no study to date has investigated the experience of meaningful coincidences and brain functional coupling of the two parts of the left IFG (i.e. pars triangularis and pars opercularis) as a central hub for cognitive control over (multi)sensory information (Tops and Boksem 2011; Li et al. 2020; Scheliga et al. 2023) and working memory content (Badre and Wagner 2007).

Based on previous work, we hypothesized that people who experience more meaningful coincidences would show alterations in the functional connectivity of the left IFG (i.e. pars triangularis or pars opercularis) with more posterior associative areas (e.g. superior parietal lobule; see Unger et al. 2021). This may indicate alterations of basic neuronal functioning in people experiencing more meaning in meaningless noise during rest (Rominger et al. 2023). To investigate this, we assessed brain activation patterns in an MRI scanner during an eyes-open resting condition. We also applied a data-driven multivoxel pattern analysis (MVPA) to our resting-state data (Nieto-Castanon 2022a) to explore whether functional connectivity of other brain areas beside the left IFG and posterior areas (e.g. superior parietal lobule) would show significant associations with meaningful coincidences. In line with previous work, we additionally hypothesized to find structural associations (i.e. reduced gray matter [GM], cortical thickness [CT], and sulcus depth [SD]) in the left IFG associated with the propensity to experience meaningful coincidences (Unger et al. 2021).

## Materials and methods

### Participants

This study is part of a larger project. We included *n* = 101 participants in the final study sample (76 women) with a mean age of 27.98 years (*SD* = 9.77). For these participants, all functional and structural MRI data as well as the coincidences questionnaire were available. All participants were right-handed and gave informed consent before participating. The ethics committee of the University of Graz approved this study (GZ. 39/4/63 ex 2022/23). Data to reproduce the reported findings are openly available (Perchtold-Stefan et al. 2024).

### The propensity to experience meaningful coincidences

Participants answered how often they perceived meaningful coincidences using seven items on a 5-point Likert scale from 1 to 5 (for all items, see Bressan 2002). Example items are “Series of clusters of names, numbers, or events of the same kind (like coming repeatedly across a word, never heard before, in the space of hours)” and “Perception of something distant in space (like worrying about a person at the exact time in which that person is having an accident).” The mean sum score of perceived meaningful coincidences was 16.59 (*SD* = 4.29). The maximum score was 35, and the minimum was 9. Analyses indicated good internal consistency with a Cronbach’s alpha of 0.79 for assessing the experience of meaningful coincidences.

### Functional and anatomical MRI data acquisition

We conducted the MRI session with a 3-T scanner (Vida; Siemens, Erlangen, Germany) with a 64-channel head coil. Blood-Oxgen-Level-Dependent (BOLD)-sensitive T2^*^-weighted functional images were acquired using an optimized multiband Echo planar imaging (EPI) (TR = 1,400 ms, TE = 30 ms, flip angle = 65°, 60 axial slices, 2.5 mm^3^ isotrop, multiband factor = 4, distance factor = 0, FoV = 220 × 220 mm^2^, interleaved slice ordering). Head motion was restricted using firm padding that surrounded the head. A total of 420 volumes were acquired while participants rested with their eyes opened, looking at a monitor displaying a gray screen (i.e. 9.8 min).

We obtained structural images using a T1-weighted Magnetization prepared rapid gradient-echo (MPRAGE) sequence (voxel size: 0.9 × 0.9 × 0.9 mm; 192 transverse slices, FoV = 224 mm, TE = 1.88 ms, TR = 1,680 ms; TI = 1,000 ms, flip angle = 8°).

### Connectivity analysis

After preprocessing of functional and structural data using fMRIPrep 23.1.3 (Esteban et al. 2019, 2023; RRID:SCR_016216), which is based on Nipype 1.8.6 (Gorgolewski et al. 2011, 2018; RRID:SCR_002502; for more details see Supplemental Information), the resulting data were analyzed via CONN toolbox (Whitfield-Gabrieli and Nieto-Castanon 2012; RRID:SCR_009550) release 22.a (Nieto-Castanon and Whitfield-Gabrieli 2022). Functional data were smoothed using spatial convolution with a Gaussian kernel of 6 mm full width at half maximum. In addition, functional data were denoised using a standard denoising pipeline (Nieto-Castanon 2020) including the regression of potential confounding effects characterized by white matter timeseries (5 CompCor noise components), cerebrospinal fluid (CSF) timeseries (5 CompCor noise components), motion parameters and their first-order derivatives (12 factors; Friston et al. 1996), outlier scans (below 247 factors; Power et al. 2014), session and task effects and their first-order derivatives (2 factors), and linear trends (2 factors) within each functional run, followed by high-pass frequency filtering of the BOLD timeseries (Hallquist et al. 2013) >0.008 Hz. CompCor (Behzadi et al. 2007; Chai et al. 2012) noise components within white matter and CSF were estimated by computing the average BOLD signal as well as the largest principal components orthogonal to the BOLD average, motion parameters, and outlier scans within each subject’s eroded segmentation masks. From the number of noise terms included in this denoising strategy, the effective degrees of freedom of the BOLD signal after denoising were estimated to range from 143.7 to 385.2 (average 357) across all subjects (Nieto-Castanon 2022b).

### Hypothesis-driven seed-to-voxel analyses

#### First-level analysis

We analyzed the seed-based connectivity maps for the resting-state condition with the CONN toolbox (Whitfield-Gabrieli and Nieto-Castanon 2012) implemented in MATLAB. For first-level analysis, the left IFG pars triangularis and pars opercularis, defined via the Harvard-Oxford atlas (implemented in the CONN toolbox; Desikan et al. 2006), served as seed areas. We calculated the temporal correlations between these two seed areas and all other voxels in the brain during rest. Functional connectivity strength was represented by Fisher-transformed bivariate correlation coefficients from a weighted general linear model (weighted-GLM; Nieto-Castanon 2020), defined separately for each pair of seed and target areas, modeling the association between their BOLD signal timeseries. To compensate for possible transient magnetization effects at the beginning of each run, individual scans were weighted by a step function convolved with an Statistical Parametric Mapping (SPM) canonical hemodynamic response function and rectified.

#### Second-level analysis

For each individual voxel, a separate GLM (Nieto-Castanon 2020) was estimated, with first-level connectivity measures at this voxel as dependent variables. We evaluated the voxel-level hypotheses using multivariate parametric statistics with random effects across subjects and sample covariance estimation across multiple measurements. Cluster-level inferences were based on parametric statistics from Gaussian Random Field theory (Worsley et al. 1996; Nieto-Castanon 2020). We used a combination of a cluster-forming *P* <0.001 voxel-level threshold and a familywise corrected p-false discovery rate (FDR) < 0.05 cluster-size threshold (Chumbley et al. 2010). We controlled for gender and age.

### Data-driven functional connectivity multivoxel pattern analysis

#### First-level analysis

We calculated functional connectivity multivariate pattern analyses (fc-MVPA) in order to evaluate if the activation of any further brain area during rest would be meaningfully related with the experience of meaningful coincidences, beside the left IFG as the seed of the theory-driven seed-to-voxel analysis (Nieto-Castanon 2022a). Significant findings would allow to identify potentially relevant target areas for future studies. We used five eigenpatterns (i.e. a conservative 20:1 ratio of participants to eigenpatterns; Westfall et al. 2020). From these eigenpatterns, five associated eigenpattern-score images were derived for each individual subject characterizing their brain-wide functional connectome state. Eigenpatterns and eigenpattern scores were computed separately for each individual seed voxel as the left- and right-singular vectors, respectively, from a singular value decomposition (group-level SVD) of the matrix of functional connectivity values between this seed voxel and the rest of the brain (a matrix with one row per target voxel, and one column per subject). Individual functional connectivity values were computed from the matrices of bivariate correlation coefficients between the BOLD timeseries from each pair of voxels, estimated using a singular value decomposition of the *z*-score normalized BOLD signal (subject-level SVD) with 64 components separately for each subject (Whitfield-Gabrieli and Nieto-Castanon 2012).

#### Second-level analysis

We performed an *F*-test on all five MVPA components. Similar to the seed-to-voxel analyses, we entered the experience of meaningful coincidences in the MVPA to determine patterns of functional connectivity associated with this measure with age and gender as covariates. Voxel-level hypotheses were evaluated using multivariate parametric statistics with random effects across subjects and sample covariance estimation across multiple measurements. Cluster-level inferences were based on parametric statistics from Gaussian Random Field theory (Worsley et al. 1996). We report results with a cluster-forming threshold of *P* <0.001 at the voxel level, and a cluster-size false discovery rate correction of p-FDR <0.05 (Chumbley et al. 2010). Following Westfall et al. (2020), we used these clusters as seeds for seed-to-voxel post hoc analysis to explore patterns of functional connectivity associated with meaningful coincidences. Since the post hoc analyses target to illustrate the direction of connectivity with the MVPA detected cluster as seed, we used a whole-brain threshold of *P* <0.005 and FDR-corrected cluster threshold of *P* <0.05 to find directional connectivity patterns for interpretations.

### Voxel-based morphometry analysis and surface analysis

#### First-level analysis

We analyzed the structural scans with Matlab R2019a(v9.6) and the Computational Anatomy Toolbox (CAT12.8.1; v1980; http://www.neuro.uni-jena.de/cat/) implemented in SPM12 (v7487; Wellcome Trust Centre for Neuroimaging; http://www.fil.ion.ucl.ac.uk/spm/software/spm12/) to gain voxel-wise comparisons of GM volume. Structural data were segmented into GM, white matter, and CSF. Spatial registration of GM images was carried out by using the optimized shooting approach (Ashburner and Friston 2011). To preserve the total amount of GM, signal images were modulated. The final resulting voxel size was 1.5 × 1.5 × 1.5 mm. Segmented GM images were smoothed with a Gaussian kernel of 8 mm full width at half maximum. Finally, only voxels with a GM volume of at least 0.1 were analyzed (absolute threshold). To examine CT, we used the surface-based morphometry approach implemented in the CAT12 toolbox. This fully automated method takes the segmented tissue classes (as already processed in the Voxel-based morphometry (VBM) analysis) and uses a projection-based algorithm to compute CT (Dahnke et al. 2013). We smoothed the vertices with an isotropic kernel of 12 mm. SD was examined with the approach implemented in the CAT12 toolbox.

#### Second-level analysis

First, we calculated a whole-brain analysis for GM volume, CT, and SD. The initial cluster building threshold was set to *P* <0.001 uncorrected, then corrected for familywise error (FWE) *P* < 0.05. To correct for differences in brain size, we implemented the total intracranial volume as a covariate (only for GM volume analysis but not for CT analyses) and used age and gender as additional covariates. For region of interest (ROI) analyses, the clusters of the connectivity analysis with left IFG as seed as well as the fc-MVPA served as specific ROI. Again, threshold was *P* <0.001 (uncorrected) followed by FWE with *P* <0.05.

## Results

### Connectivity analysis

#### Hypothesis-driven seed-to-voxel analyses (left IFG pars triangularis and pars opercularis)

**Fig. 1:**
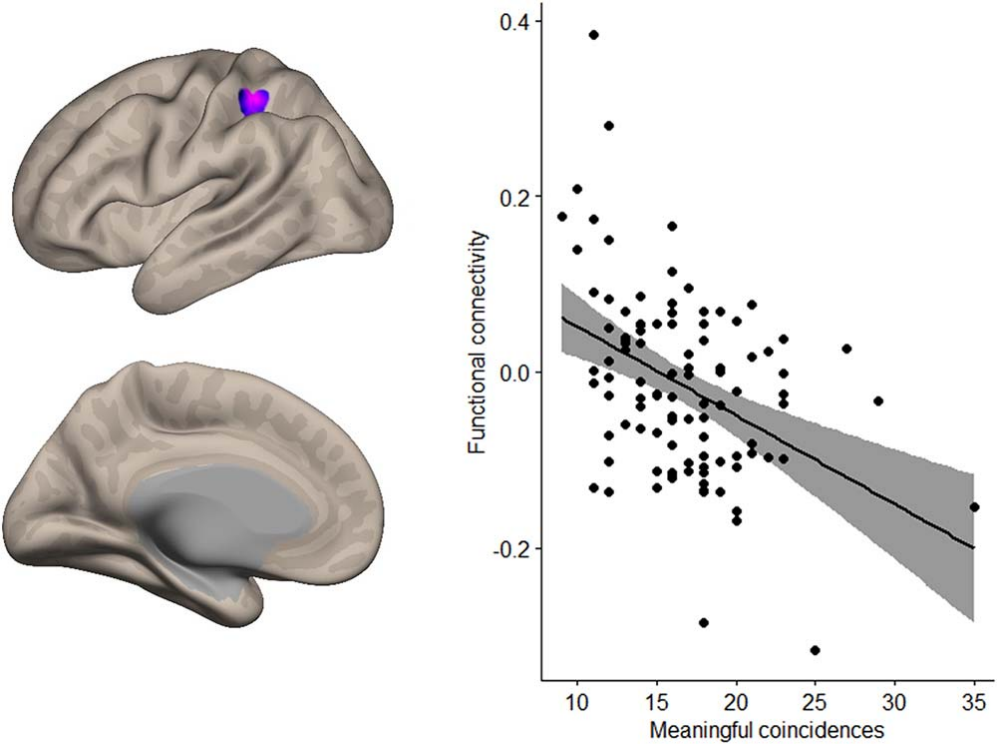
Region showing significant negative functional connectivity with the left IFG pars triangularis during resting state associated with the propensity of meaningful coincidences.

The theory-driven seed-to-voxel analysis with the left IFG (pars triangularis) as seed revealed one significant cluster, which included parts of the left superior parietal cortex, post central gyrus, and supramarginal gyrus (see Table 1). Figure 1 illustrates this cluster and the scatterplot of the correlation, which showed a medium to large effect size (*r* = −0.41). The higher the experience of more meaningful coincidences, the lower was the functional correlation between the left IFG (pars triangularis) and this specific cluster.

**Table 1:** The one cluster result of the seed-to-voxel analysis with left IFG pars triangularis as seed.

Seed	Cluster	MNI			*B*	*k*	*P*
Left IFG (pars triangularis)	Central	−34	−36	+46	−0.01	132	0.035
	Postcentral gyrus left					64	
	Superior parietal lobule left					22	
	Supramarginal gyrus					21	

*Note*. Voxel-wise threshold of level of *P* < 0.001 uncorrected and a cluster-level threshold of *P* < 0.05 FDR corrected. IFG = inferior frontal gyrus.

This result was similar when we excluded the participant with the highest score of 35 for meaningful coincidences from the analysis (see Supplemental Information, Fig. 1 and Table 1).

The seed-to-voxel analyses for the left IFG (pars opercularis) showed no significant effect.

#### Data-driven functional connectivity multivoxel pattern analysis

The fc-MVPA indicated that the pattern of connectivity between one specific cluster located in the right cerebellum and the rest of the whole brain was significantly associated with the propensity to experience of meaningful coincidences (see Table 2).

**Table 2:** Results of fc-MVPA analysis

FC regions	Brain region	MNI			*k*	*P*
MVPA		+18	−50	−22	50	0.006
	Cerebellum 6 right				25	
	Cerebellum 4 5 right				25	

Voxel-wise threshold of level of *P* <0.001 uncorrected and a cluster-level threshold of *P* <0.05 FDR corrected.

**Fig. 2:**
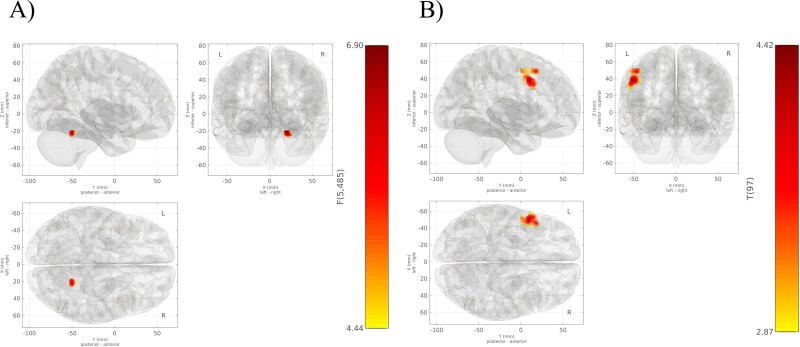
A) Whole-brain fc-MVPA results. This region shows connectivity patterns with the rest of the brain, which are significantly associated with meaningful coincidences. B) Results from the post hoc seed-to-voxel analyses for this fc-MVPA cluster.

Follow-up seed-to-voxel analyses, with this cluster located in the right cerebellum as seed region, provided a significant finding at a voxel-wise threshold level of *P* <0.005 uncorrected and a cluster-level threshold of *P* <0.05 FDR corrected (see Fig. 2).

The seed region of the fc-MVPA was correlated with a cluster located at the left hemisphere including parts of the middle frontal gyrus, precentral gyrus, as well as the left IFG (pars opercularis) as a function of meaningful coincidences (see Table 3).

**Table 3:** The one cluster result of the post hoc seed-to-voxel analysis with the fc-MVPA cluster as seed.

Seed	Cluster	MNI			*B*	*k*	*P*
MVPA	Central	−52	+10	+38	0.004	298	0.004
	Middle frontal gyrus left					169	
	Precentral gyrus left					71	
	IFG (pars opercularis) left					14	

*Note*. Voxel-wise threshold of level of *P* <0.005 uncorrected and a cluster-level threshold of *P* <0.05 FDR corrected. IFG = inferior frontal gyrus.

#### Structural correlates of meaningful coincidence

The whole-brain analysis showed no significant association between structural characteristics (i.e. GM, CT, or SD) and the experience of meaningful coincidences. Similarly, the separated ROI analyses with areas showing connectivity patterns significantly associated with meaningful coincidences (derived from the seed-to-voxel analysis and the fc-MVPA) also showed no significance for GM.

## Discussion

Why do some people more than others experience meaning in meaningless noise or have a strong feeling that their inner thoughts cause events in the outer world? This study adds further evidence to the notion that the trait to experience meaningful coincidences is associated with deviations in functional coupling of brain areas during rest. This functional coupling pattern is associated with brain areas involved in neurocognitive control mechanisms establishing working memory and sensory processing (i.e. integration of multisensory information). Both are deeply rooted in the brain of the perceivers (Rominger et al. 2019, 2023). By running a theory-driven seed-to-voxel analysis in a comparatively large sample of 101 participants, we found a higher negative functional coupling of the left IFG (pars triangularis) with left associative posterior areas such as the superior parietal cortex as well as the supramarginal gyrus.

As our analyses do not provide information about the causal direction of the observed effect, two interpretations of the negative association as well as their combination are possible. First, lower left posterior activation might lead to a higher need of the left IFG to upregulate its activation to keep working memory online and to compensate for working memory problems during the resting-state condition in the scanner. This interpretation is in accordance with the working memory hypothesis of meaningful coincidences (Hintzman et al. 1978; Diaconis and Mosteller 1989; Rominger et al. 2011). Recently, Ben-Artzi et al. (2022) suggested that people’s working memory capacities are associated with the learning of outcome-irrelevant features. During a reinforcement learning task, the authors assessed the rate at which people concluded that an irrelevant information (i.e. side of rewarded stimulus) would predict subsequent behavior. In a large sample of 174 participants, those who showed low working memory capacities tended to select more outcome-irrelevant stimuli. This demonstrates the role of working memory capacity for the tendency to assign value to irrelevant features of the environment—an important characteristic of the experience of meaningful coincidences.

Beside the left IFG (pars triangularis), which is important to inhibit irrelevant information from working memory (Badre and Wagner 2005), the superior/inferior parietal cortex in particular is a brain area relevant for manipulating information in working memory. Both areas constitute parts of the fronto-parietal network (Wager and Smith 2003; Koenigs et al. 2009), which is active when the brain establishes core executive functions such as inhibition, shifting, and updating (Rodríguez-Nieto et al. 2022). In line with this, Barredo et al. (2016) suggested that the connection between the pars triangularis part of the IFG and the parietal areas is associated with postretrieval control processes of memory such as monitoring, decision-making, and response selection (Barredo et al. 2015). This study’s functional connectivity pattern during rest therefore nicely corroborates with the working memory hypothesis of meaningful coincidences (Hintzman et al. 1978).

Alternatively, in high coincidence perceivers, the left IFG may need to downregulate posterior brain areas (i.e. superior/inferior parietal cortex, supramarginal gyrus) in order to establish normal functioning during rest and to inhibit irrelevant sensory information originating from these associative cortical areas (Rominger et al. 2023). Since the associative parietal cortex is a central hub integrating sensory information via attentional processes, irrelevant and disturbing visual sensory information flow via the dorsal path but also tactile sensations, information about limb localization, and auditory information might hamper the functioning of this complex system during the rest condition in a scanner. To conclude, since the IFG facilitates executive functioning to establish the integration of this multisensory information (Li et al. 2020; Scheliga et al. 2023), the effects of our study may indicate that the left IFG (pars triangularis) downregulates the sensory system and might stop motor actions in progress to establish normal (resting state) functioning in the scanner. This finding corresponds with the perceptual alteration hypothesis of meaningful coincidences—the higher need to inhibit the neuronal system due to a stronger environmental and sensory input in individuals experiencing more meaningful coincidences (Rominger et al. 2018, 2023). Finally, it is important to discuss that both interpretations are not contradictory, and that working memory load and sensory information processing are not independent from each other. Inhibition of irrelevant (sensory and motor) information is important to establish a well-functioning working memory system (see e.g. Jensen and Mazaheri 2010 for the gating by inhibition hypothesis).

In line with the working memory hypothesis, it seems reasonable that serial order processing is associated with individuals’ propensity to attribute significance to coincidental events. Since the intraparietal sulcus as well as the supramarginal gyrus participate during serial ordering in working memory (i.e. detecting and retaining temporal sequence; Abrahamse et al. 2014; Guidali et al. 2019; Attout et al. 2022), the decreased coupling with the left IFG (pars triangularis) might signal the hampered functioning of the left intraparietal sulcus as an attention regulator for neural networks specialized in processing sequential order (Majerus et al. 2006). Furthermore, the left IFG and left intraparietal sulcus seem to collaborate on cognitive processes essential for internal storytelling, narratives, and argumentative thoughts (Xu et al. 2021), which might highlight and emphasize the meaningful connections perceived within coincidental events.

However, it is important to note that the applied theory-driven seed-to-voxel analysis has its limits. First, we had to put a priori assumptions into the analyses and the comparatively large ROIs (i.e. left IFG pars triangularis and opercularis) consist of heterogeneous functional subregions (see e.g. Neubert et al. 2014; Barredo et al. 2016). However, due to the novelty of neuroscience of meaningful coincidences, information to select more fine-grained and smaller ROIs except the left IFG (pars triangularis and pars opercularis) was not available (see e.g. Unger et al. 2021). Second, it is important to consider potential false reverse inference when interpreting results from fMRI data (Poldrack 2006). It is not necessarily the case that the activation of a specific area indicates similar cognitive functions from one task to the other. Therefore, concluding that the activation of the left IFG (pars triangularis) indicates higher cognitive control as well as cognitive flexibility is not necessarily true. However, we would like to highlight that we carefully interpreted the present results in agreement with Poldrack by taking the applied resting-state condition and the functional connectivity of areas into account. Future studies should replicate this finding by additionally assessing behavioral data and task performance during fMRI to allow for stronger conclusions. Nevertheless, it is important to note that the present study builds on the perceptual alteration hypothesis and the working memory hypothesis, both derived from previous studies demonstrating behavioral and neurophysiological correlates (e.g. EEG activation pattern) associated with the tendency to experience meaningful coincidences (e.g. Rominger et al. 2019; Unger et al. 2021; Rominger et al. 2023).

At first glance, the present study’s functional findings seem well in accordance with the study of Unger et al. (2021). They reported decreased GM volume in the left IFG and left superior/inferior parietal cortex. However, the present study convincingly failed to find evidence for structural brain changes (i.e. GM, CT, or SD) associated with the propensity to experience meaningful coincidences. This divergence in findings of both studies may be due to the female-only sample of Unger et al. (2021) as well as further characteristics such as age and education. Since we know that age is a strong confounder of the brain’s cortical structure (e.g. gray matter volume, Christova and Georgopoulos 2023; but also white matter, Koten et al. 2023), the present sample might be more heterogeneous with respect to these variables and thus does not align with previous structural findings (Unger et al. 2021). However, we controlled for age and gender in our analyses and still did not find significant decreases in brain structure. Therefore, future studies are needed to investigate potentially moderating third variables such as gender, age, as well as education, trait emotions, and cognitive skills in larger samples to get a better understanding of the involved brain areas as well as their functional meaning for the propensity to perceive meaningful coincidences.

Besides our theory-driven functional coupling analyses, we also conducted an unbiased data-driven analysis (i.e. fc-MVPA; Nieto-Castanon 2022a). When taking the results of this fc-MVPA into account, we can conclude that beyond the functional connectivity between the left IFG pars triangularis and the left posterior associative areas, other brain regions may also play a seminal role in the perception of meaningful coincidences (at least during rest). We found that parts of the right cerebellum showed different functional connectivity with the rest of the brain when taking the experience of meaningful coincidences into account. The follow-up seed-to-voxel analysis specifically revealed a higher connectivity of the cluster in the right cerebellum with a distinct cluster including parts of the left frontal cortex (i.e. left middle frontal gyrus, left precentral gyrus, and the left IFG [pars opercularis]).

So, can the fc-MVPA add anything to the interpretation of potential reasons why people experience more meaningful coincidences? A rigorous application of this data-driven approach indicates that the right cerebellum in particular may be an interesting target area for future studies due to its importance for motor control but also cognitive functions such as attention, executive functions, and language processes (see e.g. Klein et al. 2016), all potentially relevant to perceive more meaningful coincidences. Interestingly, the positive functional connectivity of parts of the right cerebellum with the left middle frontal gyrus, the precentral gyrus, and left IFG (pars opercularis) as a function of meaningful coincidence underline these assumptions. First, the involvement of frontal areas might indicate that people with more experiences of meaningful coincidences need more executive control processes during the resting-state condition. Second, these cognitive control processes might specifically involve motor control processes during rest (indicated by left precentral gyrus and right cerebellum). Third, the right cerebellum and the left IFG are linked to semantic language processes (Nakatani et al. 2023) relevant for the experience of meaningful coincidences (see above).

When combining the findings of the theory-driven and the data-driven approaches, we can conclude that the involvement of the left IFG pars opercularis in the post hoc analysis of the fc-MVPA analysis strengthens our theoretical assumptions which put the left IFG functioning at the core of our study (Rominger et al. 2011). However, the fc-MVPA analysis did not indicate the left IFG pars triangularis itself but a network of the right cerebellum, left middle frontal gyrus, precentral gyrus, and the left IFG pars opercularis. This might indicate that the left IFG pars triangularis works fine during rest and consequently fulfills the task to downregulate or to compensate the deviated functioning of the left superior parietal cortex. Of note, our conclusions are in line with the working memory and the perceptual alteration hypothesis; however, the fc-MVPA analysis puts, beside the left IFG and the left superior parietal cortex, more emphasis on the role of the right cerebellum for experiencing meaning in meaningless noise.

## Conclusion

Although this study reports functional brain connectivity alterations in people experiencing more meaningful coincidences, it is important to recognize that these effects are subtle, and we should discuss them in a comparatively nuanced manner that focuses on individual differences instead of dysfunction. Similar to other topics in psychology, regular brain function does not operate in a manner of all-or-nothing but rather a manner of degree. In any case, brain patterns, perceptions, and cognitive functioning which seem maladaptive at the first view may actually serve adaptive trajectories as well (see e.g. Nørby 2015, 2020 for the adaptive value of forgetting). To conclude, we found significant alterations of functional connectivity of specific brain areas, arguing for deviations in control functioning relevant for sensory processing, motor control, serial order processing, and working memory associated with the experience of meaningful coincidences. However, the effects are not large enough to justify simple and overselling interpretations in terms of reduced cognitive capacities. When evaluating our findings, it is important to hold in mind that the propensity to perceive meaningful coincidences is a personality trait which also serves positive trajectories such as more everyday creative activities and more self-rated creative achievements (Rominger et al. 2024), as well as mediation (Butzer et al. 2024), and higher life satisfaction (Russo-Netzer and Icekson 2022). Following an adaptivity perspective, it is possible that reduced working memory capacities and increased sensory information flow might also increase the chance to explore and build more (loose) connections between a variety of stimuli in our environment (Ben-Artzi et al. 2022). This is one reason why some people might perceive more meaning hidden from the view of others (Carson et al. 2003; Rominger et al. 2022), which is also one prerequisite for being creative.

## Supplementary Material

SI_txt_bhae269

## Data Availability

All relevant data to reproduce the reported findings are available under https://openneuro.org/datasets/ds004965/versions/1.0.0 and https://osf.io/b2h3k/.
