# Supplementary material for: MRI resting-state signature of the propensity to experience meaningful coincidences: a functional coupling analysis
Source: Cereb Cortex. 2024 Jul 10;34(7):bhae269. doi: 10.1093/cercor/bhae269 (PMC11234293; doi:10.1093/cercor/bhae269)
Supplement: SI_txt_bhae269 [file si_txt_bhae269.docx]

**Methods**

**Data preprocessing**

Results included in this manuscript come from preprocessing performed using fMRIPrep 23.1.3 (Esteban et al. 2019; Esteban et al. 2023; RRID:SCR_016216), which is based on Nipype 1.8.6 (Gorgolewski et al. 2011; Gorgolewski et al. 2018; RRID:SCR_002502).

**Structural data**

The T1-weighted image was corrected for intensity non-uniformity (INU) with N4BiasFieldCorrection (Tustison et al. 2010), distributed with ANTs (Avants et al. 2008, RRID:SCR_004757), and used as T1w-reference throughout the workflow. The T1w-reference was then skull-stripped with a Nipype implementation of the antsBrainExtraction.sh workflow (from ANTs), using OASIS30ANTs as the target template. Brain tissue segmentation of cerebrospinal fluid (CSF), white-matter (WM) and grey matter (GM) was performed on the brain-extracted T1w using fast (FSL RRID:SCR_002823, Zhang, Brady, Smith 2001). Volume-based spatial normalization to one standard space (MNI152NLin2009cAsym) was performed through nonlinear registration with antsRegistration (ANTs), using brain-extracted versions of both T1w reference and the T1w template. The following template was selected for spatial normalization and accessed with TemplateFlow (23.0.0, Ciric et al. 2022): ICBM 152 Nonlinear Asymmetrical template version 2009c (Fonov et al. 2009, RRID:SCR_008796; TemplateFlow ID: MNI152NLin2009cAsym).

**Functional data**

We first generated a reference volume and its skull-stripped version using a custom methodology of fMRIPrep. Head-motion parameters concerning the BOLD reference (transformation matrices, and six corresponding rotation and translation parameters) were estimated before any spatiotemporal filtering using mcflirt (FSL, Jenkinson et al. 2002). The BOLD run was slice-time corrected to 0.644s (0.5 of slice acquisition range 0s-1.29s) using 3dTshift from AFNI (Cox and Hyde 1997, RRID:SCR_005927). The BOLD time-series were resampled onto their original, native space by applying the transforms to correct for head-motion. The BOLD reference was then co-registered to the T1w reference using mri_coreg (FreeSurfer) followed by flirt (FSL, Jenkinson and Smith 2001) with the boundary-based registration cost-function (Greve and Fischl 2009). Co-registration was configured with six degrees of freedom. Several confounding time-series were calculated based on the preprocessed BOLD: framewise displacement (FD), DVARS and three region-wise global signals. FD was computed using two formulations following Power et al. (2014; absolute sum of relative motions) and Jenkinson et al. (2002; relative root mean square displacement between affines). FD and DVARS were calculated for each functional run, both using their implementations in Nipype (following Power et al. 2014). The three global signals were extracted within the CSF, the WM, and the whole-brain masks. Additionally, a set of physiological regressors were extracted to allow for component-based noise correction (CompCor, Behzadi et al. 2007). Principal components were estimated after high-pass filtering the preprocessed BOLD time-series (using a discrete cosine filter with 128s cut-off) for the two CompCor variants: temporal (tCompCor) and anatomical (aCompCor). tCompCor components are then calculated from the top 2% variable voxels within the brain mask. For aCompCor, three probabilistic masks (CSF, WM, and combined CSF+WM) are generated in anatomical space. The implementation differs from that of Behzadi et al. (2007) in that instead of eroding the masks by 2 pixels on BOLD space, a mask of pixels that likely contain a volume fraction of GM is subtracted from the aCompCor masks. This mask is obtained by thresholding the corresponding partial volume map at 0.05, and it ensures components are not extracted from voxels containing a minimal fraction of GM. Finally, these masks are resampled into BOLD space and binarized by thresholding at 0.99 (as in the original implementation). Components are also calculated separately within the WM and CSF masks. For each CompCor decomposition, the *k* components with the largest singular values are retained, such that the retained components’ time series are sufficient to explain 50% of variance across the nuisance mask (CSF, WM, combined, or temporal). The remaining components are dropped from consideration. The head-motion estimates calculated in the correction step were also placed within the corresponding confounds file. The confound time series derived from head motion estimates and global signals were expanded with the inclusion of temporal derivatives and quadratic terms for each (Satterthwaite et al. 2013). Frames that exceeded a threshold of 0.5 mm FD or 1.5 standardized DVARS were annotated as motion outliers. Additional nuisance timeseries were calculated employing principal components analysis of the signal found within a thin band (crown) of voxels around the edge of the brain, as proposed by Patriat, Reynolds, Birn (2017). The BOLD time series were resampled into standard space, generating a preprocessed BOLD run in MNI152NLin2009cAsym space. First, a reference volume and its skull-stripped version were generated using a custom methodology of fMRIPrep. All resamplings can be performed with a single interpolation step by composing all the pertinent transformations (i.e., head-motion transform matrices, susceptibility distortion correction when available, and co-registrations to anatomical and output spaces). Gridded (volumetric) resamplings were performed using antsApplyTransforms (ANTs), configured with an interpolation to minimize the smoothing effects of other kernels (Lanczos 1964). Non-gridded (surface) resamplings were performed using mri_vol2surf (FreeSurfer).

**Results**

The one cluster result of the seed-to-voxel analysis with left IFG pars triangularis as seed after excluding one participant with the highest score for meaningful coincidences. Voxel wise threshold of level of *p*<.001 uncorrected and a cluster-level threshold of *p*<.05 FDR corrected.

| Seed | Cluster | MNI |  |  | B | k | p |
| --- | --- | --- | --- | --- | --- | --- | --- |
| Left IFG (pars triangularis) | Central | -38 | -36 | +36 | -0.01 | 130 | .04 |
|  | Postcentral Gyrus Left | |  |  |  | 55 |  |
|  | Superior Parietal Lobule Left | |  |  |  | 13 |  |
|  | Supramarginal Gyrus |  |  |  |  | 39 |  |

Note. IFG = inferior frontal gyrus


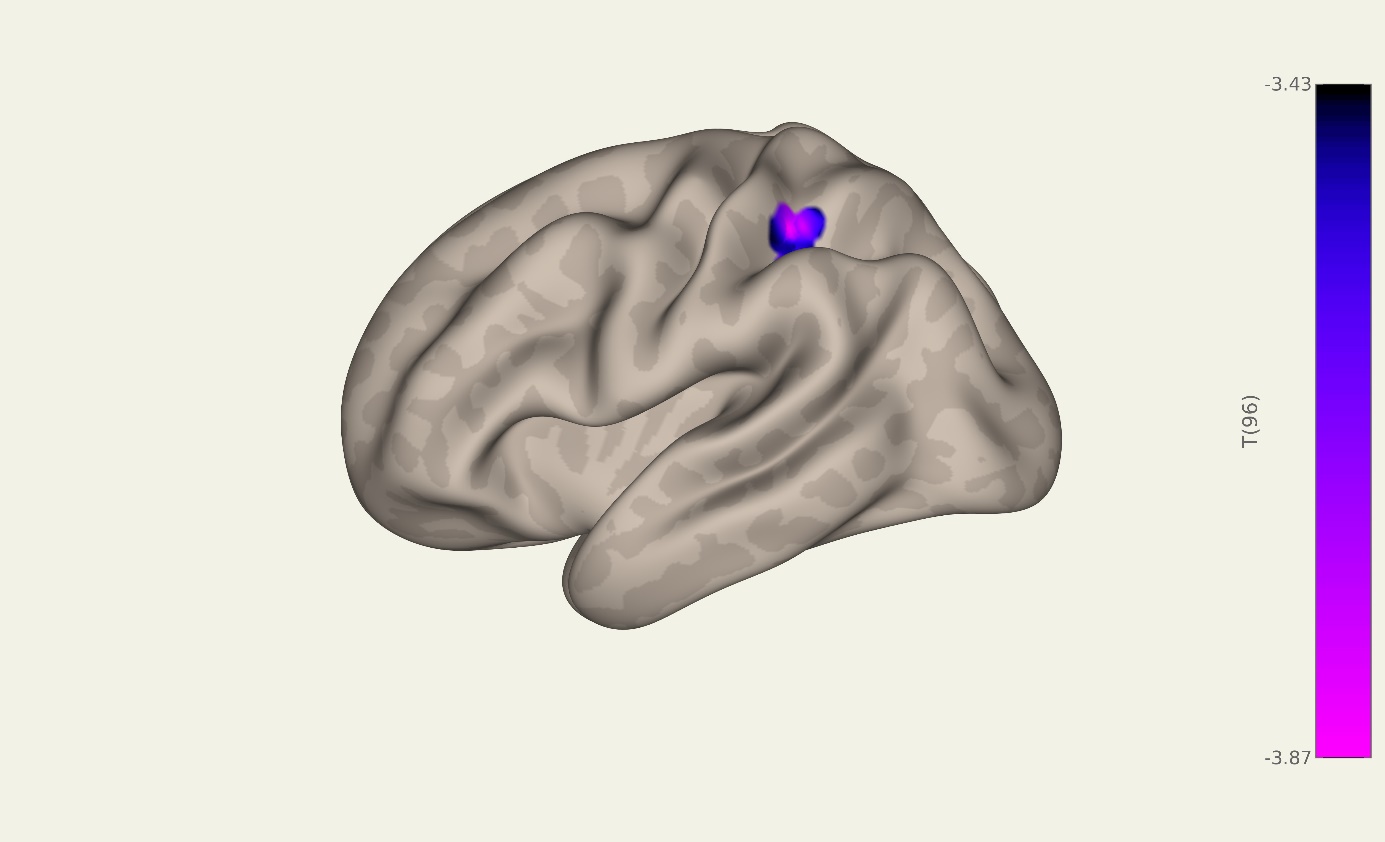


**Figure 1.** Region showing significant negative functional connectivity with the left IFG pars triangularis during resting state associated with the propensity of meaningful coincidences after excluding one participant with the highest score of 35 for meaningful coincidences.

**References**

Avants BB, Epstein CL, Grossman M, Gee JC. 2008. Symmetric diffeomorphic image registration with cross-correlation: Evaluating automated labeling of elderly and neurodegenerative brain. Medical Image Analysis. 12(1):26–41.

Behzadi Y, Restom K, Liau J, Liu TT. 2007. A component based noise correction method (CompCor) for BOLD and perfusion based fMRI. NeuroImage. 37(1):90–101.

Ciric R, Thompson WH, Lorenz R, Goncalves M, MacNicol EE, Markiewicz CJ, Halchenko YO, Ghosh SS, Gorgolewski KJ, Poldrack RA, et al. 2022. TemplateFlow: FAIR-sharing of multi-scale, multi-species brain models. Nature Methods. 19(12):1568–1571.

Cox RW, Hyde JS. 1997. Software tools for analysis and visualization of fMRI data. NMR Biomed. 10(4-5):171–178.

Esteban O, Blair R, Markiewicz C, Berleant S, Moodie C, Ma F, Isik A, Erramuzpe A, Kent J, Goncalves M, et al. 2023. poldracklab/fmriprep: 23.1.3. Zenodo.

Esteban O, Markiewicz CJ, Blair RW, Moodie CA, Isik AI, Erramuzpe A, Kent JD, Goncalves M, DuPre E, Snyder M, et al. 2019. fMRIPrep: A robust preprocessing pipeline for functional MRI. Nature Methods. 16(1):111–116.

Fonov V, Evans A, McKinstry R, Almli C, Collins D. 2009. Unbiased nonlinear average age-appropriate brain templates from birth to adulthood. NeuroImage. 47:S102.

Gorgolewski KJ, Burns CD, Madison C, Clark D, Halchenko YO, Waskom ML, Ghosh SS. 2011. Nipype: A flexible, lightweight and extensible neuroimaging data processing framework in python. Frontiers in Neuroinformatics. 5:13.

Gorgolewski KJ, Esteban O, Markiewicz CJ, Ziegler E, Ellis DG, Jarecka D, Notter MP, Johnson H, Burns C, Manhães-Savio A, et al. 2018. nipy/nipype: 1.1.3: Zenodo.

Greve DN, Fischl B. 2009. Accurate and robust brain image alignment using boundary-based registration. NeuroImage. 48(1):63–72.

Jenkinson M, Bannister P, Brady M, Smith S. 2002. Improved optimization for the robust and accurate linear registration and motion correction of brain images. NeuroImage. 17(2):825–841.

Jenkinson M, Smith S. 2001. A global optimisation method for robust affine registration of brain images. Medical Image Analysis. 5(2):143–156.

Lanczos C. 1964. Evaluation of noisy data. Journal of the Society for Industrial and Applied Mathematics Series B Numerical Analysis. 1(1):76–85.

Patriat R, Reynolds RC, Birn RM. 2017. An improved model of motion-related signal changes in fMRI. NeuroImage. 144(Pt A):74–82.

Power JD, Mitra A, Laumann TO, Snyder AZ, Schlaggar BL, Petersen SE. 2014. Methods to detect, characterize, and remove motion artifact in resting state fMRI. NeuroImage. 84:320–341.

Satterthwaite TD, Elliott MA, Gerraty RT, Ruparel K, Loughead J, Calkins ME, Eickhoff SB, Hakonarson H, Gur RC, Gur RE, et al. 2013. An improved framework for confound regression and filtering for control of motion artifact in the preprocessing of resting-state functional connectivity data. NeuroImage. 64:240–256.

Tustison NJ, Avants BB, Cook PA, Zheng Y, Egan A, Yushkevich PA, Gee JC. 2010. N4ITK: Improved N3 bias correction. IEEE Transactions on Medical Imaging. 29(6):1310–1320.

Zhang Y, Brady M, Smith S. 2001. Segmentation of brain MR images through a hidden Markov random field model and the expectation-maximization algorithm. IEEE Transactions on Medical Imaging. 20(1):45–57.
